# Evaluation of the Impacts of a Phone Warning and Advising System for Individuals Vulnerable to Smog. Evidence from a Randomized Controlled Trial Study in Canada

**DOI:** 10.3390/ijerph16101817

**Published:** 2019-05-22

**Authors:** Kaddour Mehiriz, Pierre Gosselin

**Affiliations:** 1 Doha Institute for Graduate Studies, School of Public Administration and Development Economics, P.O. Box: 200592, Zone 70, Al Tarfa Street Al-Daayen, Doha, Qatar; 2 Institut National de la Santé Publique and Ouranos, 945 Avenue Wolfe, Québec, QC G1V 5B3, Canada; Pierre.gosselin@inspq.qc.ca

**Keywords:** automated phone smog warning system, smog episodes, impact evaluation, randomized controlled trial design, climate change adaptation, Canada, smog vulnerability

## Abstract

Smog warning systems are components of adaptation strategies that are adopted by governments around the world to protect their citizens from extreme episodes of air pollution. As part of a growing research stream on the effectiveness of these systems, this article presents the results of a study on the impacts of an automated phone warning and advising system for individuals vulnerable to air pollution. A sample of 1328 individuals were recruited and randomly assigned to treatment and control groups. The treatment group received smog warning while the control group did not. Data were collected via three phone surveys, two before and one after issuing the smog warning. The comparison between treatment and control groups indicates that exposure to a smog warning improved information on the occurrence of smog episodes (*n* = 484, OR = 5.58, *p* = 0.00), and knowledge on protective behaviors. Furthermore, members of treatment group were more likely to avoid exposure to smog episodes by spending more time inside with the windows closed than usual (*n* = 474, OR = 2.03, *p* = 0.00). Members of treatment group who take medication in the form of aerosol pumps also kept these devices on themselves more frequently than those of control group (*n*= 109, OR = 2.15, *p* = 0.03). The system however had no discernible effects on the awareness of air pollution risks, reduction of health symptoms related to smog and the use of health system services. The absence of health benefits could be related to the lower actual exposure to air pollution of such vulnerable groups during winter.

## 1. Introduction

Smog episodes are severe air pollution periods characterized by a mixture of smoke and fog resulting from natural and/or anthropogenic factors [1]. The frequency and intensity of smog episodes is presumed to increase with global warming and urbanization, which represents a major concern for public health authorities, notably in the current context marked by an increase in the proportion of seniors that are particularly vulnerable to this hazard [2,3,4]. According to a recent report of the World Health Organization (WHO), air pollution is already the biggest environmental threat to population health as it is the cause of approximatively 10% of deaths [5]. In addition to seniors, individuals with cardiovascular and respiratory diseases as well as children are more at risk of suffering from this hazard [6].

Efforts to curb greenhouse gas emissions are presently at the core of governments’ strategies to contain global warming as evidenced by the Paris Agreement on Climate Change [7]. In addition to mitigation plans, governments also rely on adaptation strategies to limit the impacts of air pollution on population health such is the case of the Quebec’s Government Action Plan on Climate Change 2013–2020 [8]. As part of their adaptation strategies, many countries have implemented air quality monitoring and warning systems [9,10,11,12]. These systems are designed to issue alerts when pollution reaches levels representing significant risk to population health [13]. In addition to providing information on the occurrence of smog episodes, alerts are accompanied by advice to reduce the risks and consequences of exposure to air pollution [12]. The underlying assumption of smog warning systems is that the exposure to alerts improves information on the occurrence of smog episodes, their risks as well as on protective behaviors. This improvement would then lead to adopting recommended behaviors and, ultimately, mitigating the adverse effects of smog on population health and reducing health services use [7,10].

In parallel to the implementation of air quality warning systems, interest in the evaluation of their performance has increased in recent years. In accordance with the logic model of smog warning systems, special attention was paid to their impacts on the adoption of protective behaviors [14,15,16], reduction in air pollution related morbidity and mortality [17] and use of health system services [11,12]. In addition, a variety of impact evaluation methods have been used, including the regression discontinuity design [17,18], quasi-experimental design [11,12] and self-reported outcomes [6,13]. The results of these studies show that air quality warnings improve adherence to protective behaviors and that the magnitude of this effect is influenced by psychological factors such as the perception of air pollution levels and risks [19]. However, evidence of smog alerts effects on morbidity and mortality, and on the use of health systems is mixed. Chen et al. [17] found a reduction in the number of emergency admissions as a result of the implementation of an air quality program in Ontario (Canada), but no effect on smog related mortality like cardiovascular and respiratory-related deaths. Likewise, McLaren and Williams found that air quality alerts were not correlated with the number of daily hospital admissions [12]. Finally, the Lyon and collaborators study shows that exposure to air pollution alerts even resulted in a substantial increase in hospital admissions, a quite unexpected outcome considering that reduction of pressure on the health system is among the main goals of these warning and advice measures [11].

The objective of this study is to evaluate the impacts of an automated phone smog warning system (APWS) for individuals vulnerable to this hazard. Its contribution to this field of research is two-fold. First, it is so far among the rare if not the only study using an experimental design to assess the effects of smog warnings. As it is well known, randomization improves significantly the internal validity of impact evaluations. Second, while most studies analyzed the effects of alerts using mass media, the focus of this study is on the effects of automated phone warnings. APWSs have the advantage of enabling the delivery of personalized smog alerts and advice to targeted individuals while avoiding overcrowding public medias with these warnings [20].

The structure of this article is as follows. In the next section, the study’s material and methods are presented. The Section 3 is devoted to study findings. This is followed by the discussion of these findings and a conclusion.

## 2. Material and Methods 

The evaluation of the APWS is based on an experimental design in which a sample of study participants were voluntarily recruited and randomly assigned to treatment and control groups (see below). The APWS was programmed to issue automated phone smog warnings along protective advice to treatment group when Environment Canada predicts that the level of air pollution will reach levels considered prejudicial to population health. To assess its impacts, data on outcome variables were collected when the actual level of air pollution was equal or higher than the threshold triggering smog warnings (i.e., a true alert). Comparisons between treatment and control on outcome variables was used to assess the effects of this system. A detailed description of the design of this experiment is provided in the following sections.

Ethics Certification: The Protocol of This Study Was Approved by the Ethics Committee of the Institut National DE La Recherche Scientifique: Cer-15-370.2.1.

### 2.1. The Design of the Intervention 

The APWS was developed and tested between 2015 and 2017 by the Institut National de Santé Publique du Québec (INSPQ) and the Direction Régionale de la Santé Publique de la Montérégie (DSP Montérégie). It is part of Quebec government efforts to deal with climate change challenges facing this province. The APWS was designed to inform vulnerable individuals on the occurrence of excessive heat and smog episodes and provide them with advice on how to protect themselves from these hazards. Compliance with the recommended behaviors is presumed to mitigate the adverse effects of smog on health and reduce health services use. 

The results on the impacts of heat warnings were published in a previous issue of this journal [20]. This article thus presents the findings related to the second component of this research project, i.e., the evaluation of the effects of winter smog warnings. Winter smog episodes occur in Canada as a result of using fossil fuel and wood heating systems in periods of low levels of atmospheric dispersion (Government of Canada [21]) and from usual industrial and transportation sources. These episodes are associated with an excess of morbidity and mortality mostly for individuals suffering from respiratory and cardiovascular diseases [10].

The APWS was designed to issue warning alerts when the forecasted air pollution reaches levels representing a serious threat to the participants in this study. Regarding this, Environment Canada uses Air Health Quality Index (AHQI) to monitor air quality across different regions of Canada [22]. AHQI is calculated based on the relative risk combination of ozone at the ground level (O_3_), particulate matter (PM_2.5_/PM_10_) and nitrogen dioxide (NO_2_). To facilitate the communication of health pollution risks to the population, the index was divided into four levels of health risks: low, moderate, high and very high risk. Environment Canada issues air quality warnings on its website when the predicted risk level is considered moderate, high or very high. These alerts are then disseminated by mass media in Canada such as TV and radio channels. 

The APWS was programmed to issue smog warnings along with protective tips when Environment Canada forecasts that the risk level of air pollution is moderate or higher on the AHQI’s scale. This level of triggering smog alerts was chosen because the objective of the APWS is to serve individuals that are particularly vulnerable to air pollution. Specifically, the APWS was tested on a sample of individuals having at least one of the following characteristics that, according to scientific literature and a committee of experts formed specifically to advise the authors of this study, are associated with vulnerability to heatwaves and air pollution [10]:

- Be 65 years old or older;
- Present a heart or lung medical condition;
- Suffer from diabetes, kidney failure, mental health disorder or neurological disorder

The smog warning message was developed in consultation with experts in public health and after reviewing the relevant scientific publications (in the study of McLaren and Williams for a summary of health advice [12]). It was then pretested on a small group of individuals vulnerable to smog in order to evaluate its relevance and clarity. In the final version of the message, recipients are advised to adopt the following behaviors during smog episodes:

- Stay inside as much as possible and close windows.
- Outside, avoid physical efforts, even the short health walks.
- If you take medication in the form of aerosol pumps, always keep them with you.
- For any questions about your health, call Info-Santé at 811.
- In case of chest pain or difficulty breathing, call 911.

The APWS was programmed to issue automatically the prerecorded oral smog alert on the usual landline or mobile phone numbers of treatment group members before the onset of smog episodes. The automated phone message was not modulable according to the severity of smog episodes or vulnerability of study participants. 

The research team decided to test the performance of the APWS in the winter rather than in the summer season. The reason being that heat waves are frequently accompanied with smog episodes and therefore some advice provided to avoid smog exposure (like stay inside the windows closed), conflicts with tips intended to protect from heat (open the windows at night; frequent cool areas such as pools and beaches). 

### 2.2. Questionnaire Design 

Phone surveys were used to collect data on participant characteristics such as gender, age, level of education and outcome. In addition, the questionnaire included questions measuring the main outcomes of the APWS.

#### 2.2.1. Improvement of Information on the Occurrence of Smog Episodes, Relevant Adaptation Strategies and Risk Perception

Two questions were used to determine whether respondents were informed or not about the smog episode. The first question asked respondents whether they were aware of the episode or not; the second, the date when they became aware of it, i.e., before, during or after smog episode. Data on participants’ knowledge of protective behaviors was collected via an open-ended question on the best ways of protecting themselves from the smog. The number of measures cited by respondents that matched the recommended behavior was then calculated for each respondent. The perception of the adverse effects of smog on health was measured with a five-level scale: 1: smog is not dangerous at all to 5: smog is extremely dangerous to my health. 

#### 2.2.2. Adoption of Recommended Behaviors

As mentioned earlier, recipients of the warning message were advised by the APWS to adopt the following behaviors during smog episodes: stay indoors with the windows shut, avoid intense outside physical effort and, for individuals using pump devices for respiratory medication, keep these devices always with them. A five-level scale was used to collect information on weather respondents stayed indoor the windows shut longer or less than usual during smog episode (1: much less than usual to 5: much longer than usual). Regarding physical efforts, respondents were first asked if they had made, or not, intense outdoor physical efforts during the smog episode. Those who responded affirmatively were then asked to rate on a five-level scale the length of this activity (1: much less than usual to 5: much longer than usual). Similarly, individuals who take respiratory medication were asked to report on a four-level scale the extent to which they kept the pump devices with them (1: never to 4: all the time).

#### 2.2.3. Mitigation of Health Symptoms Related to Smog and Use of the Health System Services

Respondents had to report if they suffered from any of the following symptoms caused by air pollution during the smog episode or within the following two or three days: difficulty in breathing, chest pain, cough and eye irritation. A dichotomous variable was created that takes the value of 1 if an individual reported that he suffered at least one of these symptoms and 0 otherwise. Regarding the use of health services, respondents were asked to indicate whether, during the smog episode or the following two or three days, they had called a nurse, pharmacist or doctor; called 811 (Info Health, personalized info by nurses); were hospitalized; visited an emergency room; consulted a doctor or nurse at a clinic; or consulted a pharmacist. A dichotomous variable was then created taking the value of 1 if the respondent used any of these services and 0 otherwise.

The first draft of the questionnaire was reviewed by a panel of five public health experts and pretested on 22 individuals filling the admissibility criteria mentioned above. In addition, the questionnaire was tested by the survey firm for items clarity on a sample of study participants before the beginning of data collection.

### 2.3. Participants Recruitment, Groups Formation and Data Collection

A sample of 1328 participants vulnerable to smog were recruited in this study from the city of Longueuil, Canada in 2015 and randomly assigned to treatment and control groups (for more details on the recruitment process, see Mehiriz et al. [20]). Participants were fully informed, when agreeing to participate, that they would be randomly assigned to these groups. To avoid between groups contamination, individuals with the same phone number were randomly assigned to the same group. Among the 1328 participants, 662 formed the treatment group and 666 control group.

Data on the effects of smog warnings were collected through three phone surveys. The first survey was conducted immediately after participants recruitment, from 25 June to 14 July 2015, to obtain data on the socio-economic and demographic characteristics of all study participants. A response rate of 76% was obtained. The purpose of the second survey was collecting baseline data on the variables measuring the effects of smog alerts. It took place after the smog alert issued by Environment Canada for 8 January 2016. A total of 770 interviews were conducted, which corresponds to a 76.3% response rate. As the objective of this survey was to obtain ex ante measures, no smog warning was issued by the APWS on that date. Environment Canada also issued a smog alert in Longueuil area on its website for the period of 5 and 6 March 2016. In accordance with the protocol of this study, a smog alert was sent by the APWS to the treatment group members only. Data on the effects of this alert was then collected through the third survey that took place between 7 and 8 March 2016. A total of 519 interviews were conducted, which corresponds to a response rate of 67.4%. Data on participants who were outside the Longueuil region during the smog episodes were not collected because the smog warnings were less relevant to their situation. 

**Figure 1:**
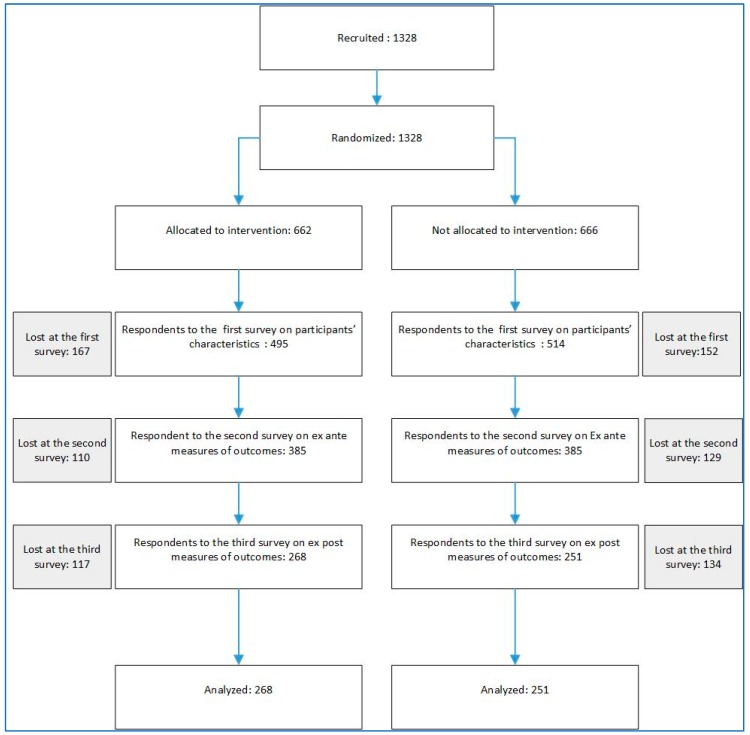
Consort Chart for automated phone smog warning system (APWS) experiment.

The Consort chart of this experiment is presented in Figure 1

### 2.4. Data Analysis 

Frequencies were used in this study to describe the distributions of binary and ordinal variables. Odds ratios were also used for these variables to compare treatment and control groups and then estimate the impacts of exposure to smog warnings. The odds ratios were obtained by running binary and ordered logistic regressions in STATA (StataCorp LP., College Station, TX, USA). 

A continuous variable was used to measure participants’ knowledge of coping strategies. A *t*-test of means difference for independent samples was then used to estimate the effect of smog alert on this variable. 

## 3. Study Results

### 3.1. Sample Characteristics and Baseline Differences

Table 1 on sample characteristics indicates that most participants are women, senior individuals and persons with chronic medical conditions. The comparison between treatment and control groups shows the absence of statistically significant differences except for annual household income where the proportion of individuals with less than 25,000 dollars is slightly higher in the treatment group.

Table 2 provides baseline data on the outcomes measured using binary and ordinal variables. Data were collected after the first smog episode during which the APWS did not issue any alert to treatment group members. It indicates the absence of statistically significant differences between treatment and control groups for all the outcome variables of this table. The two groups were also equivalent regarding the number of smog protection measures cited (mean difference = 0.02; *p* = 0.46).

Data on sample characteristics and baseline differences suggest that the randomization process ensured the equivalence between the treatment and control groups and, as a result, there was no need for further controls of preexisting differences.

### 3.2. Impacts of the APWS 

Data on the effects of the APWS were collected after the smog alert of 7 March and 8 March 2016. The main results are summarized in Table 3, Table 4 and Table 5. 

#### 3.2.1. Effect on Information on the Occurrence of Smog Episodes, Knowledge about Coping Strategies, and Risk Perception

Table 3 shows that half of the participants (49.7%) were informed on the occurrence of the smog episode. We noticed also a strong difference between the experimental and control groups. The proportion of individuals who were informed on the occurrence of the smog episode amounts to 70.5% in the case of the first group against 30.1% for the second group. As indicated in Table 4, this difference corresponds to an odds ratio of 5.58. 

We observed also a slight difference between treatment and control groups regarding the number of protection measures cited. Members of the experimental group cited an average of one out of three recommended behaviors against 0.91 for the control group (*p* = 0.01).

Table 5 indicates that 41.1% of respondents consider that smog is somewhat dangerous to their health, which suggests a moderate perception of smog risks. In addition, the perception of risk level is not affected by exposure to the APWS’ smog alerts as the odds ratio between treatment and control groups is not statistically significant (OR = 1.15, *p* = 0.40). This result could probably be explained by the fact that the warning message did not provide information on smog risks. 

#### 3.2.2. Adoption of Recommended Behaviors

Concerning respondents’ behavior during the smog episode, 80% of the respondents reported that they stayed indoor the windows closed as much as usual (Table 5). Experimental group members, however, were more likely to adhere to this advice compared to control group (OR = 2.03, *p* = 0.00). They also kept medication on them more often than usual comparatively to the control group (OR = 2.15, *p* = 0.03). 

The analysis of the APWS’ effect on physical efforts shows the absence of differences between the two groups (OR = 0.59, *p* = 0.22). This advice seems to be less relevant for the participants in this study that, given their health conditions, only a very small proportion of them (5%) made intense physical efforts during winter smog episodes (Table 3).

#### 3.2.3. Mitigation of Health Symptoms Related to Smog and the Use of the Health System

Data on study participants indicate that approximatively 30% of respondents suffered smog related symptoms and 6.5% used health system services during the second smog episode (Table 3). The comparison between the experimental group and control groups suggest that smog alerts did not have an impact on these variables in this context. As shown in Table 4, the odds ratio of the first variable is equal to 1.05 (*p* = 0.81) and that of the second variable is equal to 1.03 (*p* = 0.92). 

This study suggests that the APWS allow recipients to be better informed on the occurrence of smog episodes and improve their knowledge about coping strategies. Smog alerts also seem to increase significantly compliance with two of the three recommended behaviors. The APWS however does not seem to raise awareness of smog risks, alleviate the symptoms related to this hazard and reduce the use of the health system. 

## 4. Discussion

An APWS was used in this study to reach vulnerable groups and inform them about the occurrence of smog episodes and coping strategies. Recipients of smog warnings are supposed to be more informed on the occurrence and risks of smog episodes and have better knowledge on how to protect themselves comparatively to non-recipients. Furthermore, the improvement of information and knowledge is expected to encourage adherence to recommended behaviors which, in turn, results in the reduction of risks of suffering from smog symptoms and of using health system services.

The results of this study indicate that automated phone warnings significantly improve information on the occurrence of smog episodes. As mentioned before, the APWS was programmed to send smog alerts to treatment group when Environment Canada issues a smog warning on its website. This substantial difference between the treatment and control groups can thus be considered as measuring the additional effect of the APWS to the effects of the smog warning system already in place in Canada, generally available through the media; at the time of the study it was not available through text or email messages, although it became available very recently. 

The baseline data of this study suggest that the current Canadian smog warning system has a low capacity of reaching vulnerable groups as only 30% of the respondents were informed of the first smog warning issued by Environment Canada. This is concordant with the results of a 2015 survey in Hamilton, Canada showing that 60% of the respondents were aware of the existence of the AHQI system and only 27% checked it [14]. This low level of coverage should be considered seriously because, contrary to heat waves, smog episodes are difficult to detect by our senses only. The population depends on sophisticated monitoring and warning systems to obtain reliable and timely information on air quality and adapt its behavior consequently. There is thus a real need for developing new smog warning methods that go beyond the simple dissemination of alerts through mass media. UK for instance has implemented an air quality alert system that allows subscribers to receive information on air quality via text, phone call, email or internet [12]. With this regard, our study provides evidence supporting the idea that automated phone warning systems seem to be a promising solution to improve the reach of smog alerts for vulnerable subgroups.

This study also indicates that automated phone warning improves adherence to recommended measures of coping with smog episodes, thus confirming the findings of previous studies on this subject. For instance, Wen and Mokdad [16] found that poor air quality alerts result in the reduction of outdoor activities among people with asthma. Likewise, a review of 21 studies by D’Antoni and al. [19] found evidence supporting the idea that exposure to air quality alerts improves compliance with recommended behaviors. However, as the cost of intertemporally substituting activities increases overtime, adherence to smog protective behaviors is likely to decrease with time [15]. This eventuality raises concerns about the performance of warning systems during long smog episodes, as is frequently the case in several countries’ metropolitan areas. 

This study suggests that the APWS warnings failed to mitigate health symptoms associated with poor air quality as well as to reduce the use of health system services. This finding nurtures the uncertainty surrounding the health benefits of smog warnings in general. In a population-based cohort study, Chen and al. [17] show that the implementation of an air quality alert program in Ontario (Canada) was associated with some reductions in respiratory morbidity, but not with the other health outcomes examined. Lyon and al. [11] even found that smog warnings have the adverse effect of increasing hospital admissions for respiratory conditions as well as emergency department attendance. The authors suspect that this unexpected outcome could be attributed to the fact that warning messages frequently advise recipients to consult health professionals if they suffered smog related symptoms. This seemingly nonexistent or, at best, small contribution of smog alerts to the mitigation of health problems invites to rethink seriously the role of smog warnings systems. Chen et al. [17] call for enforced public actions to reduce air pollution instead of relying on smog alerts only. As is the case of the APWS, some alert systems are implemented with the purpose of protecting senior individuals and those with chronic medical conditions. However, members of this target population do not spend a long time in outdoor activities comparatively to other social groups and, as a result, are often less exposed to smog episodes [15]. The baseline data of our study also show that only 6% of participants reported making outside intense physical efforts during the first smog episode. Given this low-level of risk exposure, improvement in the compliance with the recommended behaviors would not have much effect on the reduction of symptoms related to smog and, therefore, on the use of the health system. Therefore, it seems thus health benefits of smog warnings would be more significant if they targeted individuals with intense and long periods of outdoors activities such as construction and road workers, and sportsmen and sportswomen.

The result of this study suggests that the APWS does not increase recipients’ awareness of the adverse effects of smog. Risk perception has been found to be an important determinant of the adoption of protective behaviors [19]. The performance of the APWS could thus be improved by including relevant information on the negative impacts of exposure to smog on population health. Such information may improve recipients’ awareness and, therefore, compliance with recommended behaviors. 

This study presents some limitations. While the warning system is intended to protect vulnerable groups from smog episodes in general, the scope of this study remains relative to the impacts of winter smog warnings. We should thus be cautious about the generalization of this study findings as the participants in this experiment, given their health conditions, are presumed to substantially reduce their outdoor activities in the winter. They therefore have low exposure to winter air pollution episodes comparatively to those of the summer season. The conclusions are also based on a single two-day smog episode which could limit its generalization potential. Finally, it should be reminded that participants in this study were not randomly selected from a defined population; this could also affect the generalization of findings. 

## 5. Conclusions

We used in this study an experimental design to measure the effects of an automated phone warning system on individuals vulnerable to smog. The comparison between treatment and control groups shows that exposure to smog warning improves information on the occurrence of smog episodes. Treatment group members also have more knowledge on how to protect themselves from this hazard. They are likewise more likely to adopt the recommended behaviors than members of the control group. The analysis however shows that the system has no discernible effect on the awareness of smog health risks, reduction of symptoms related to smog as well as on the use of health system. The low risk of target population exposure to smog may explain the absence of beneficial health effects of smog warning.

## Figures and Tables

**Table 1:** Sample characteristics.

Variable	Treatment		Control		*p*-Value
	*n*	%	*n*	%	
Cardiovascular disease	492	51.0	508	52.9	0.54
Bronchial or lung disease	491	22.6	507	22.5	0.96
Diabetes	489	19.4	509	20.0	0.81
Kidney failure	488	4.9	509	4.9	1.00
Neurological diseases	487	7.8	510	7.3	0.84
65 years or over	495	87.1	511	86.3	0.87
Women	479	74.5	500	75.8	0.65
Elementary school diploma	487	15.6	502	12.2	0.12
Secondary school diploma	487	32.9	502	34.1	0.69
College diploma	487	20.9	502	19.7	0.63
University degree	487	30.6	502	34.1	0.24
Household income (Thousands $): Less than 25	395	38.1	394	31.1	0.04
Between 25 and 50	395	33.3	394	36.2	0.38
Between 51 and 75	395	17.5	394	18.5	0.72
More than 75	395	11.2	394	14.2	0.20

*n*: Number of respondents to each question. (Source: Mehiriz et al. [20])

**Table 2:** Comparison between treatment and control groups before exposure to phone smog warning message.

Outcomes	*n*	Odds Ratio	Confidence Interval	*p*-Value
Were informed of smog episode	754	0.94 (0.07)	0.80–1.10	0.45
Perception of smog vulnerability	752	0.94 (0.06)	0.82–1.06	0.34
Stayed indoor more (or less) than usual	738	1.00 (0.07)	0.82–1.23	0.94
Made physical efforts	741	1.00 (0.15)	0.74–1.36	0.99
Frequency of keeping medication on him/herself	162	0.88(0.13)	0.66–1.17	0.37
Suffered smog related symptoms	769	0.87 (0.07)	0.75–1.01	0.07
Used health system	771	0.86 (0.11)	0.67–1.12	0.27

**Table 3:** Ex post measurement of outcomes measured by binary variables.

Outcomes	Treatment		Control	
	*n*	%	*n*	%
Were informed of smog episode	241	70.5%	246	30.1%
Average number of smog protection measures cited	244	1	251	0.91
Made physical efforts	243	3.7%	245	6.1%
Suffered smog related symptoms	244	30.7%	251	29.9%
Used health system services	244	6.6%	251	6.4%

**Table 4:** Differences between treatment and control groups after exposure to the APWS smog warning.

Outcomes	*n*	Odds Ratio	Confidence Interval	*p*-Value
Were informed of smog episode	484	5.58 (1.11)	3.78–8.23	0.00
Perception of smog vulnerability	469	1.15 (0.19)	0.82–1.60	0.40
Stayed indoors more or less than usual	474	2.03 (0.48)	1.28–3.24	0.00
Made physical efforts	485	0.59 (0.25)	0.25–1.38	0.22
Frequency of keeping medication on him/herself	109	2.15 (0.78)	1.06–4.37	0.03
Suffered smog related symptoms	492	1.05 (0.20)	0.71–1.54	0.81
Used health system services	492	1.03 (0.38)	0.51–2.12	0.92

**Table 5:** Ex post measurement of outcomes measured on ordinal scales.

Outcomes	*n*	1	2	3	4	5
Perception of smog vulnerability (1: not dangerous at all, 5: extremely dangerous)	496	15.5%	18.6%	41.1%	21.4%	3.4%
Stayed indoors longer or less than usual (1: much less than usual, 5: much longer than usual)	500	1.8%	1.8%	80%	10.2%	6.2%
Frequency of keeping medication on him/herself (1: never, 4: all the time)	114	25.4%	6.1%	22.8%	45.6%	NA
